# The psychoactive effects of *Bryophyllum pinnatum* (Lam.) Oken leaves in young zebrafish

**DOI:** 10.1371/journal.pone.0264987

**Published:** 2022-03-09

**Authors:** Kassia Martins Fernandes Pereira, Ana Calheiros de Carvalho, Thiago André Moura Veiga, Adam Melgoza, Raúl Bonne Hernández, Simone dos Santos Grecco, Mary Uchiyama Nakamura, Su Guo

**Affiliations:** 1 Department of Obstetrics, Universidade Federal de São Paulo, São Paulo, SP, Brazil; 2 Department of Bioengineering and Therapeutic Sciences, Programs in Biological Sciences and Human Genetics, University of California, San Francisco, San Francisco, California, United States of America; 3 Department of Chemistry, Universidade Federal de São Paulo, Diadema, SP, Brazil; 4 Pharmaceutical Sciences and Pharmacogenomics Graduate Program, University of California, San Francisco, San Francisco, California, United States of America; 5 Laboratory of Bioinorganic and Environmental Toxicology–LABITA, Department of Chemistry, Universidade Federal de São Paulo. Diadema. SP. Brazil; 6 Triplet Biotechnology Solutions, São Paulo, Brazil; Monash University Malaysia, MALAYSIA

## Abstract

*Bryophyllum pinnatum* (Lam.) Oken (BP) is a plant that is used worldwide to treat inflammation, infections, anxiety, restlessness, and sleep disorders. While it is known that BP leaves are rich in flavonoids, the extent of the beneficial and toxic effects of its crude extracts remains unclear. Although some neurobehavioral studies using leaf extracts have been conducted, none has examined the effects of water-extracted leaf samples. The zebrafish is a powerful animal model used to gain insights into the efficacy and toxicity profiles of this plant due to its high fecundity, external development, and ease of performing behavioral assays. In this study, we performed behavioral testing after acute exposure to different concentrations of aqueous extract from leaves of *B*. *pinnatum* (LABP) on larval zebrafish, investigating light/dark preference, thigmotaxis, and locomotor activity parameters under both normal and stressed conditions. LABP demonstrated dose-and time-dependent biphasic effects on larval behavior. Acute exposure (25 min) to 500 mg/L LABP resulted in decreased locomotor activity. Exposure to 300 mg/L LABP during the sleep cycle decreased dark avoidance and thigmotaxis while increasing swimming velocity. After sleep deprivation, the group treated with 100 mg/L LABP showed decreased dark avoidance and increased velocity. After a heating stressor, the 30 mg/L and 300 mg/L LABP-treated groups showed decreased dark avoidance. These results suggest both anxiolytic and psychoactive effects of LABP in a dose-dependent manner in a larval zebrafish model. These findings provide a better understanding of the mechanisms underlying relevant behavioral effects, consequently supporting the safe and effective use of LABP for the treatment of mood disorders.

## Introduction

The *Bryophyllum pinnatum* (Lam.) Oken (BP) or *Kalanchoe pinnata* (Lam.) Pers. (KP) [1] is a plant belonging to the Crassulaceae family, more popularly known as “life-plant,” “fortune-plant,” “bruja” (Caribbean), “saião,” “coirama,” or “folha-de-pirarucu” (Brazil). Although it originates from Madagascar, it is commonly found in tropical regions worldwide [2, 3]. This species is known for its resistance to drought for long periods and is easy to reproduce because its leaves sprout easily from edges. The leaves extract of BP (LBP) is primarily composed of cinnamic acids and flavonoids. Pereira *et al*. [4] identified the following LBP components through HPLC/MS/MS: cinnamic acids–caffeic and coumaroylglutaric acids; organic acids–malic, citric, and isoctric acids; and flavonoids–cyanidin-3-glucoside, dihydroquercetin, glucosyl arabinosyl rhamnosyl quercetin, isorhamnetin hexose pentose, miricetin-3-O-rhamnoside (miricitrin), quercetin 3- O-α-L-arabinopyranosyl-(1→2)-α-L-rhamnopiranoside, quercetin-3- O-α-L-rhamnopiranosíde (quercitrin), kaempferol 3-O-α-L-arabinopyranosyl -(1→2)-α-L-rhamnopyranoside (kapinnatoside), and isoscoparin-2”-O- arabinoside. In addition, LBP have demonstrated *in vitro* anticancer activity [4]. The leaves are used in infusions, juices, and pastes in traditional medicine to treat inflammations and infections [5]. Additionally, its tincture and press juice forms are used to treat anxiety and sleep disorders and prevent preterm delivery [6].

Flavonoids, which have been identified as a component of LBP, are natural compounds with antioxidant potential as well as neuroprotective, anxiolytic, sedative, and anticonvulsant properties [7]. These effects on the central nervous system (CNS) are mediated by different interactions with enzymes, receptors, and signaling pathways, especially GABAa receptors [7]. Although LBP have traditionally been used to treat anxiety, sleep disorders, and other conditions [5], the characterization of their behavioral effects and stress effectiveness remains unclear.

The zebrafish (ZF; *Danio rerio*) is an excellent model with many applications and advantages owing to its wide range of observable phenotypes, such as physiology, metabolism, and behavior [8]. The ZF has optical transparency, a small size, high fecundity, and low housing costs [9]. Around 3–4 days post-fertilization, larval stage ZF complete embryonic development and become free-swimming with measurable patterns of behavior; after around 2–3 months, ZF reach sexual maturity and become adults [9]. Furthermore, ZF can easily be exposed to chemicals via simple immersion, where the drug is rapidly absorbed through the gills and body surface, although it should be noted that our understanding of ZF pharmacokinetics is limited and the exact concentrations of chemicals absorbed into cells are still being explored [10]. Importantly, 84% of disease-associated genes in humans have ZF orthologues [9]. Thus, neuroanatomical and genetic similarities, along with accessibility and a rich repertoire of behaviors such as social activity, anxiety, learning, and memory, suggest that ZF could be used to study processes related to human disease and CNS development [9, 11]. Neurobehavioral effects can be analyzed by quantifying locomotor and swimming behaviors in ZF after exposure to chemical substances, and the results are similar to those observed in mammals [12]. Moreover, neurobehavioral events can be quantified to examine fear/anxiety-like behavior. Fear and anxiety are innate emotional states that trigger defensive responses in situations that are potentially harmful to human health. However, when these responses manifest excessively, they can cause a clinical condition known as an anxiety disorder. Behaviors of escape and avoidance are associated with fear and anxiety in animals [13]. Light/dark preference and thigmotaxis (position near the wall) are parameters used to measure fear/anxiety-like behavior, a behavioral trait related to optimal survival and fitness in both ZF and mammals. ZF larvae innately show a baseline level of dark avoidance, or light preference, and a preference for edges of the chamber over the center zone, or center avoidance. Increased trends in these behaviors may be indicative of fear and anxiety states [14].

Most previous *in vitro* and *in vivo* studies of the effects of LBP used ethanol or methanol extracts in mice or rats, and anxiety-like behavior was rarely assessed [5, 15–18]. Methanol and ethanol are common vehicles for dissolving drugs administered to experimental organisms, but by themselves, they can alter physiology and behavior at high concentrations [19, 20]. Hot aqueous extracts, known as tea, are simple and inexpensive to prepare, making them the most popular form of natural product consumption, being widely consumed by humans, including pregnant women and children, because of their calming and mood-regulating properties [5]. Furthermore, this simple aqueous formulation of LBP allows for the study of its toxicological and behavioral profiles, excluding the potential effects of other solvents.

Therefore, the objective of this study was to verify the precise mechanisms of the behavioral changes associated with LABP in ZF. The chemical composition of LABP was evaluated using an ultra-efficiency liquid chromatography coupled with a mass spectrometer (UHPLC/DAD/HRMS-MS/MS), followed by behavioral analyses in ZF larvae after acute exposure to the plant extract, during the sleep cycle, and after heating and sleep deprivation stressors. We verified that LABP decreased avoidance and increased exploratory behavior after different periods of exposure to normal and stressful situations. These data provide valuable information with implications for safe and effective natural therapeutic options for behavioral and emotional disorders in humans.

## Methods

### Plant material, extract, and sample preparation

Leaves from BP were collected in August 2018 in the city of São Roque, interior of the state of São Paulo, Brazil. Botanical identification was carried out by the Instituto Agronômico (IAC) linked to Secretaria de Agricultura e Abastecimento do Estado de São Paulo, in which a voucher specimen was deposited under number 129/2013. A copy of the voucher specimen was also deposited at the Succulent Collection at the Zurich Botanical Garden, Switzerland (number ZSS 29717).

The LABP extract was prepared by incubating fresh leaves in boiling water for 15 min, and then subjecting the tea to lyophilization to obtain the lyophilized aqueous extract from *Bryophyllum pinnatum—*LABP.

### Chemical analysis

The LABP was chemically characterized using a Shimadzu Ultra Efficiency Liquid Chromatograph in conjunction with a Bruker high-resolution QToF mass spectrometer (UHPLC/DAD/HRMS-MS/MS). For separation, a Kinetex RP_18_ analytical column from Phenomenex (Kinetex 2.6μ, 100x2,1MM, 2.6 μm of particle size) was used as the stationary phase at 55°C, and a multi-step gradient eluotropic system, composed of ultrapure water (A) and acetonitrile spectroscopic grade (B), both with 0.1% formic acid. The wavelength detector was set for reading in the scanning mode in the range of 190–800 nm. The sample was prepared at a concentration of 1 mg/mL, diluted with LBP extract in ultrapure water, and filtered through a PTFE filter, Chromafil Xtra GF-100/25 (Macherey-Nagel Gmbh & Company KG). The sample injection volume was 10 μL. The mass spectrometer was operated in both the positive and negative ionization modes. The temperature of the drying gas was 200°C at a flow of 9 Lmin-1, 2 bar for the nebulizer pressure, and 4500 V of capillary voltage (kV). Sodium formate was used as the calibrator. Fragments were selected from well-resolved chromatographic bands with a mass-to-charge ratio (m/z) of 50–1200 Da. The data obtained were analyzed using DataAnalysis 4.4 software (Bruker). The parameters for processing mass accuracy were below 8 ppm and mSigma less than 50.

### Animals and house

ZF larvae (*D*. *rerio*) used for the experiments were obtained from the AB strain. Pairs of AB wild-type fish (a male and a female) were placed in the same partitioned aquarium overnight. The following morning, the divider was removed to allow the animals to cross. After a few hours, the eggs were collected and sorted into separate 100 mm Petri dishes filled with BEW (approximately 35 eggs/ 40 mL/ dish). These embryos were incubated at 28°C for 2 days post fertilization (dpf), and at 3 dpf, the dishes were transferred to the ZF facility and exposed to a regular circadian cycle (14:10 h light/dark cycle) at 28°C. At the end of the experiments, fish were euthanized by rapid submersion in ice water for a minimum of ten minutes after cessation of all opercular movements. All procedures were approved by the Institutional Animal Care and Use Committee of the University of California San Francisco (UCSF-IACUC, approval number: AN179000-02) and the Ethics Committee on the Use of Animals from the Faculty of Medicine of the University of São Paulo (CEUA-FMUSP–approval number: n° 1312/2019). Animal experiments were carried out with humane care according to institutional guidelines.

### *Danio rerio* embryo test

Collection of eggs and exposure of embryos were performed as described by Hernández *et al*. [21]. Ten embryos were exposed to each concentration in 96-well microplates. Each well received one embryo and 200 mL of exposure medium. Static exposure was performed during different periods of development, that is, from 2 to 50 hours post-fertilization (hpf), 24–72 hpf, 72–122 hpf, and 2–120 hpf. Embryos were exposed to medium with LABP (1–3000 mg/mL) or without (control group). Lethality was identified by coagulation of the embryo, missing heartbeat, failure to develop somites, or non-detached tail. All exposure experiments were performed at least in triplicate, using batches of embryos from different breeding tanks.

### Behavioral testing (BT)

LABP was diluted in blue egg water (BEW) consisting of 0.12 g CaSO_4_, 0.2 g Instant Ocean Salts from Aquatic Eco-systems, 30 μL methylene blue in 1 L of H_2_O, on the same day as the test to obtain sample solutions with different concentrations. LABP doses used were 1, 3, 100, and 300 mg/L for 20 min and 500 mg/L for 25 min and 60 min for behavioral testing after acute exposure, 100 and 300 mg/L for sleep cycle exposure, 30 mg/L for the heat stressor test, and 100 mg/L for sleep deprivation.

The light/dark choice assay was used to verify the effects of LABP. In the light/dark choice assay, larval ZF were found to prefer light (or avoid dark), and this behavior is thought to be anxiety-related. Thus, the increased percentage of time spent on the light side in larval ZF is believed to reflect increased anxiety. Behavioral tests were conducted using larvae at 6 dpf for acute exposure and heat stressor experiments and 7 dpf for sleep deprivation and sleep cycle experiments and were performed in the diurnal period (between 9 am and 3 pm).

#### Light/dark choice assay apparatus

A lightbox was laid on a table with incandescent light bulbs placed on each side of the lightbox, illuminating the compartment with diffuse light. Black and white stripes, 5 cm wide and made from infrared transmitting acrylic (ACRYLITE IR acrylic 11460), were then affixed to the top of the lightbox. The light intensity of the light side was 2000 lx, and that of the dark side was 50 lx. Each light/dark test apparatus consisted of four individual square chambers (4 cm × 4 cm × H1.5 cm for each).

Each chamber was divided into two compartments of equal size: one white and one black. The sides of the compartments were surrounded by opaque white and black tape to eliminate wall transparency. Each chamber was filled with 10 ml blue egg water at 28°C (water depth: ~5 mm).

### LABP acute exposure

To investigate the behavioral effects of acute LABP exposure, larvae were transferred to a behavioral room 30 min before the experiment and then sorted in Petri dishes filled with LABP solutions at doses of 1, 3, 30, 100, 300, and 500 mg/L for different periods of exposure (20, 25, and 60 min). Approximately 40 larvae were analyzed *per* dose. After acute exposure, behavioral testing was performed as described previously.

### LABP exposure during the sleep cycle

For sleep cycle experiments, larvae (6 dpf) were kept in the dark in the presence of BEW (control), 100, or 300 mg/L LABP during the sleep cycle (10 pm–8 am), using black tape to cover the dishes in a behavior room, and then the light/dark choice assay was conducted. Behavioral testing was performed at 7 dpf as previously described.

### Sleep deprivation testing

For the sleep deprivation test, the light/dark choice assay was performed after the 6 dpf larvae had been kept on the light in the presence of 100 mg/L LABP during the sleep cycle (10 pm-8 am), using a lightbox. Behavioral testing was performed at 7 dpf as previously described.

### LABP exposure in the presence of a heat stressor

For the stressor test, a light/dark choice assay was performed using heat as a stressor stimulus. Larvae (6 dpf) were added to dishes containing 10 mL of BEW (control) or 30 mg/L and 300 mg/L LABP at 50°C or room temperature. Behavioral testing was performed after 5 min of introducing the heat stressor. Behavioral testing was performed as previously described.

### Video tracking, data, and statistical analysis

Sixteen light/dark square chambers were simultaneously recorded during the behavioral test. An 8-min recording was made using a camera positioned above the tanks. The videos were recorded using Noldus MPEG Recorder 2.1 as digital video files. At the end of the 8-min recording, larval ZF were gathered in a dish and euthanized with ice. Digital video files and video track acquisitions were analyzed using EthoVision XT 13. The output parameters included swimming velocity and distance, time spent in the light and dark sides to calculate the choice index, and time spent in the inner and outer zones of the arena, with the same area drawn in Ethovision for thigmotaxis.

The following formula was used for the choice index analysis: choice index = (duration in dark zone − duration in light zone)/total time. Thigmotaxis analysis was performed using the following formula: Thigmotaxis % = [(duration in the inner zone − duration in the outer zone)/total time] × 100.

Data were visualized using GraphPad Prism (version 5.00). Statistical analyses included ANOVA followed by the unpaired t-test for two comparisons (i.e., between the LAPB sample and the control group) and Dunnett’s and Sidak’s multiple comparisons tests. Values were considered significant at p<0.05, with a 95% confidence interval.

## Results

### Chemical composition of LABP extracts

**Fig 1:**
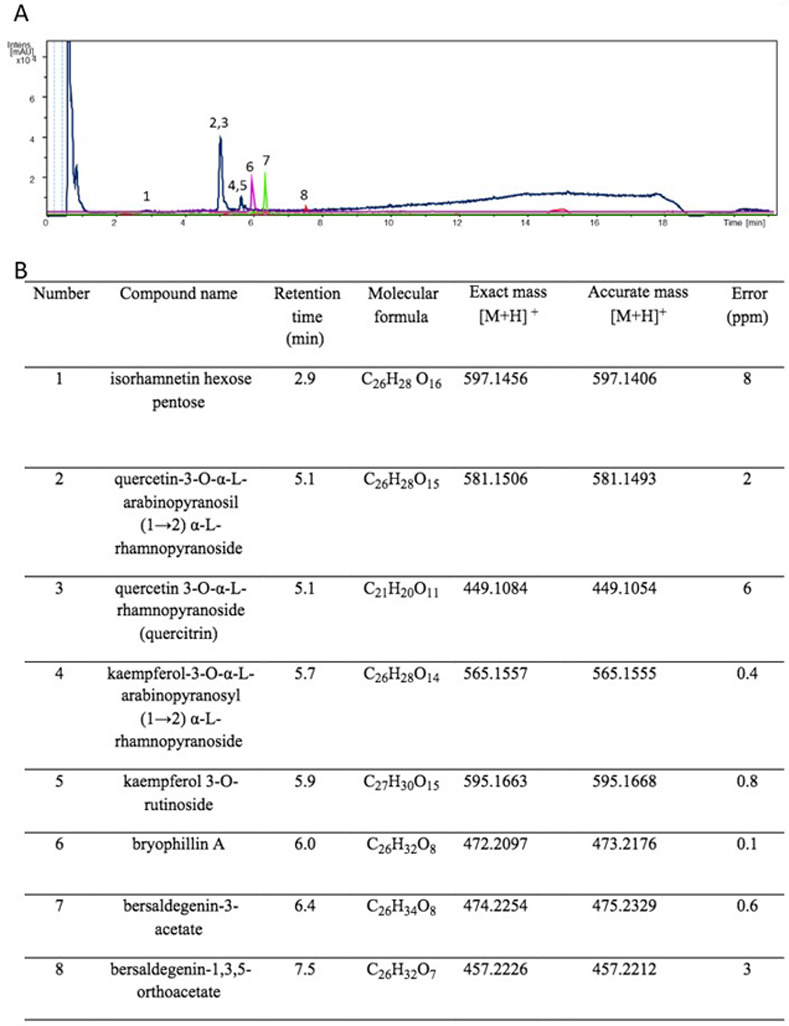
LABP chemical profile characterization. **A.** Overlapping of UV (ultraviolet) and EIC (extracted ions) chromatograms obtained from analysis of LABP analyzed by UPLC/DAD-ESI/HRMS/MS (electrospray ionization—positive ion mode). The blue line corresponds to the UV chromatogram. The purple, green and red lines correspond to the EIC. **B.** The table shows the compounds annotated following the peaks, retention times, molecular formulas, and masses.

LABP extract was analyzed by UPLC/DAD-ESI/HRMS/MS to determine its chemical profile and ensure the quality of the extract (Figs 1 and S1). Based on *the m/z* values and fragmentation patterns, in comparison with previously described compounds for the species, it was possible to annotate five glycosylated flavonoids (**1–5**): isorhamnetin hexose pentose (**1**), quercetin-3-O-α-L-arabinopyranosil (1→2) α-L-rhamnopyranoside (**2**), quercetin 3-O-α-L-rhamnopyranoside (quercitrin) (**3**), kaempferol-3-O-α-L-arabinopyranosyl (1→2) α-L-rhamnopyranoside (**4**), and kaempferol 3-O-rutinoside (**5**). These five compounds can be seen in the UV chromatogram (Fig 1A; peaks 1–5, blue line). Through extracted ion chromatogram (EIC) analysis, it was also possible to annotate three bufadienolides (**6–8**): bryophillin A (**6**), bersaldegenin-3-acetate (**7**), and bersaldegenin-1,3,5-orthoacetate (**8**) (Fig 1B), consistent with previous literature [22].

### Toxicity of LABP in ZF embryos

Toxicity assays revealed that only 120 h with high doses of LABP exposure resulted in significant larval death. Larvae exposed to 2146 mg/L and 524 mg/L LABP caused 50% and 5% of the deaths, respectively (S2 Fig). Consequently, LABP concentrations lower than 524 mg/L could have a safe pharmacological effect and were used in subsequent behavioral studies.

### Acute exposure to LABP affects exploratory and anxiety-like behaviors in a dose-dependent manner

**Fig 2:**
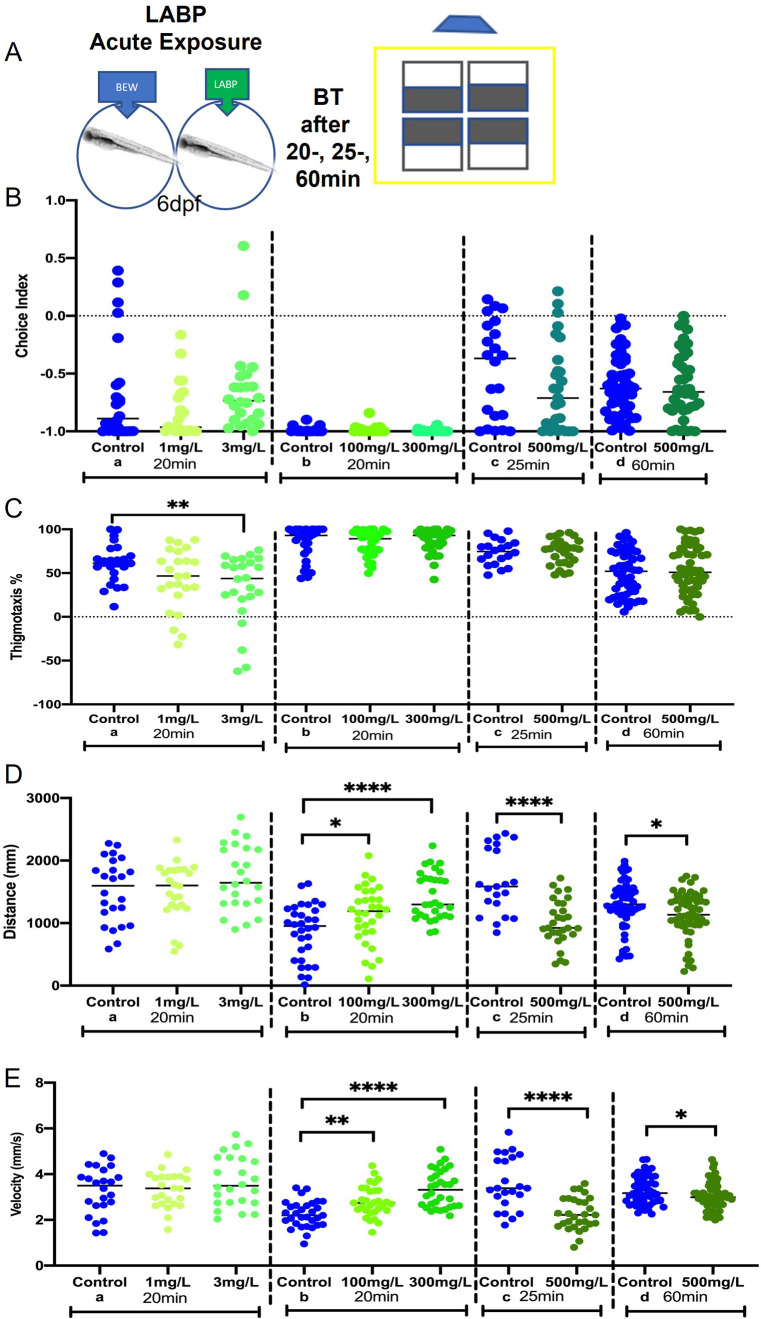
Behavior effects after LABP acute exposure. **A.** The schematic of the behavioral testing after LABP exposure (20, 25, or 60 min) in different concentrations (1, 3, 100, 300, or 500 mg/L). **B.** There was no significant difference in choice index between the control group and LABP treated groups. **C.** After 20 min of 3 mg/L LBP exposure decreased thigmotaxis significantly (p = 0.0082). The other groups did not show significant differences when compared to control. **D.** 100 and 300 mg/L LABP added 20 min before testing increased distance significantly (p = 0.0297 and p<0.0001, respectively). 500 mg/L LABP decreased the distance significantly after 25- and 60-min exposure both (p<0.0001 and p = 0.0285, respectively). **E.** 100 and 300 mg/L LABP added 20 min before testing increased velocity significantly (p = 0.0031 and p<0.0001, respectively). 500 mg/L LABP decreased velocity significantly after both 25- and 60-min exposure (p<0.0001 and p = 0.0254, respectively). Asterisks indicate statistical differences between groups (*p < 0.05, **p < 0.01, ***p < 0.001, ****p < 0.0001). n = 24/group a-20 min; 28/group b-20 min; 28/group c-25 min; 50/group d-60 min.

To investigate the effects of acute LABP exposure on exploratory and anxiety-like behaviors, the larvae were exposed to different concentrations of LABP (1, 3, 100, 300, or 500 mg/L) for 20, 25, or 60 min (Fig 2).

The choice index was not significantly different between the control and LABP groups in all experiments. However, a trend toward an increase in the choice index was observed in larvae treated with 3 mg/L LABP after 20 min of exposure (p = 0.6736) (Fig 2B). Thigmotaxis significantly decreased in the group treated with 3 mg/L LABP (p = 0.0082) (Fig 2C). The distance and velocity were affected in a dose-dependent manner (Fig 2D and 2E). While 100 and 300 mg/L LABP significantly increased distance (p = 0.0297 and p<0.0001, respectively) and velocity (p = 0.0031 and p<0.0001, respectively) after 20 min exposure, 500 mg/L LABP decreased the same parameters after 25- and 60-min exposure (distance, p<0.0001 and p = 0.0285, respectively; velocity, p<0.0001 and p = 0.0254, respectively).

### LABP exposure during the sleep cycle affects exploratory and anxiety-like behaviors

**Fig 3:**
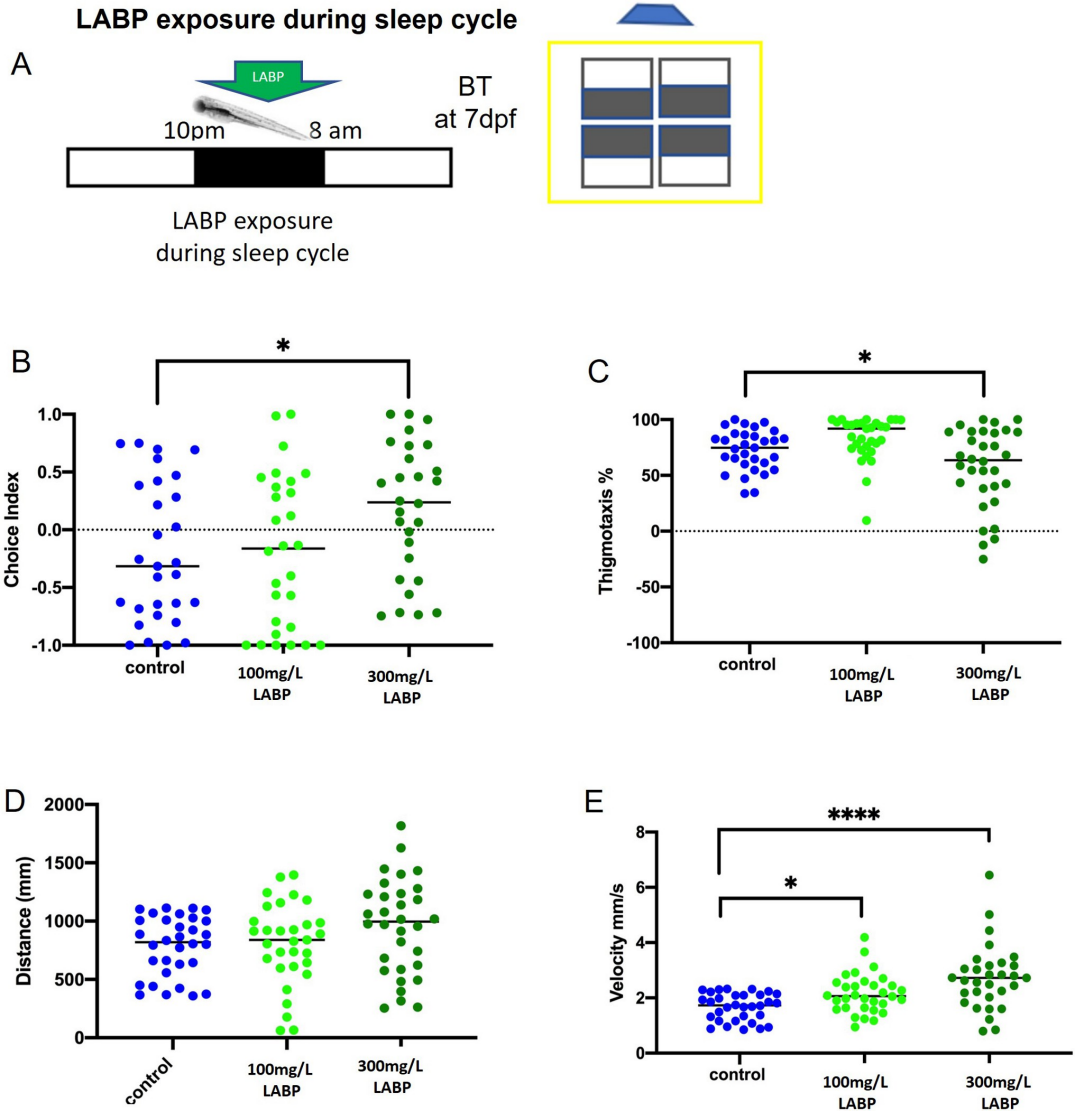
Behavior effects after LABP exposure during the sleep cycle. **A.** The schematic of the behavior test after LABP exposure during the sleep cycle. The larvae were kept on dark and LABP exposure from 10 pm-8 am. **B.** 300 mg/L LABP significantly increased the choice index when compared to control (p = 0.0410). **C.** Thigmotaxis significantly showed a reduction in the group treated with 300 mg/L LABP when compared to the control group (p = 0.0346). **D.** Distance was increased in 300 mg/L LABP but not significantly (p = 0.0870). **E.** LABP significantly increased velocity in both 100 and 300 mg/L concentrations (p = 0.0416; p<0.0001). Asterisks indicate statistical differences between groups (*p < 0.05, **p < 0.01, ***p < 0.001, ****p < 0.0001). n = 30/group.

To determine whether LABP exposure during the larval sleep cycle could affect behavior the next morning, 6 dpf larvae were kept in the dark in LABP solution during sleep hours, 10 pm-8 am (Fig 3), and behavioral testing was performed at 7 dpf (Fig 3A). Both dark avoidance and thigmotaxis were significantly decreased when ZF larvae were exposed to 300 mg/L LABP compared to the control groups (p = 0.0410 and p = 0.0346, respectively) (Fig 3B and 3C). After exposure to 100 and 300 mg/L LABP, swimming velocity was significantly increased compared to the control (p = 0.0416 and p<0.0001, respectively) (Fig 3E), and the distance traveled in the 300 mg/L group showed an increasing trend but did not reach statistical significance (p = 0.0870) (Fig 3D).

### LABP exposure relieves stressor-aggravated anxiety-like behaviors

#### Light-induced sleep deprivation increases dark avoidance and decreases velocity that can be abrogated by LABP exposure

**Fig 4:**
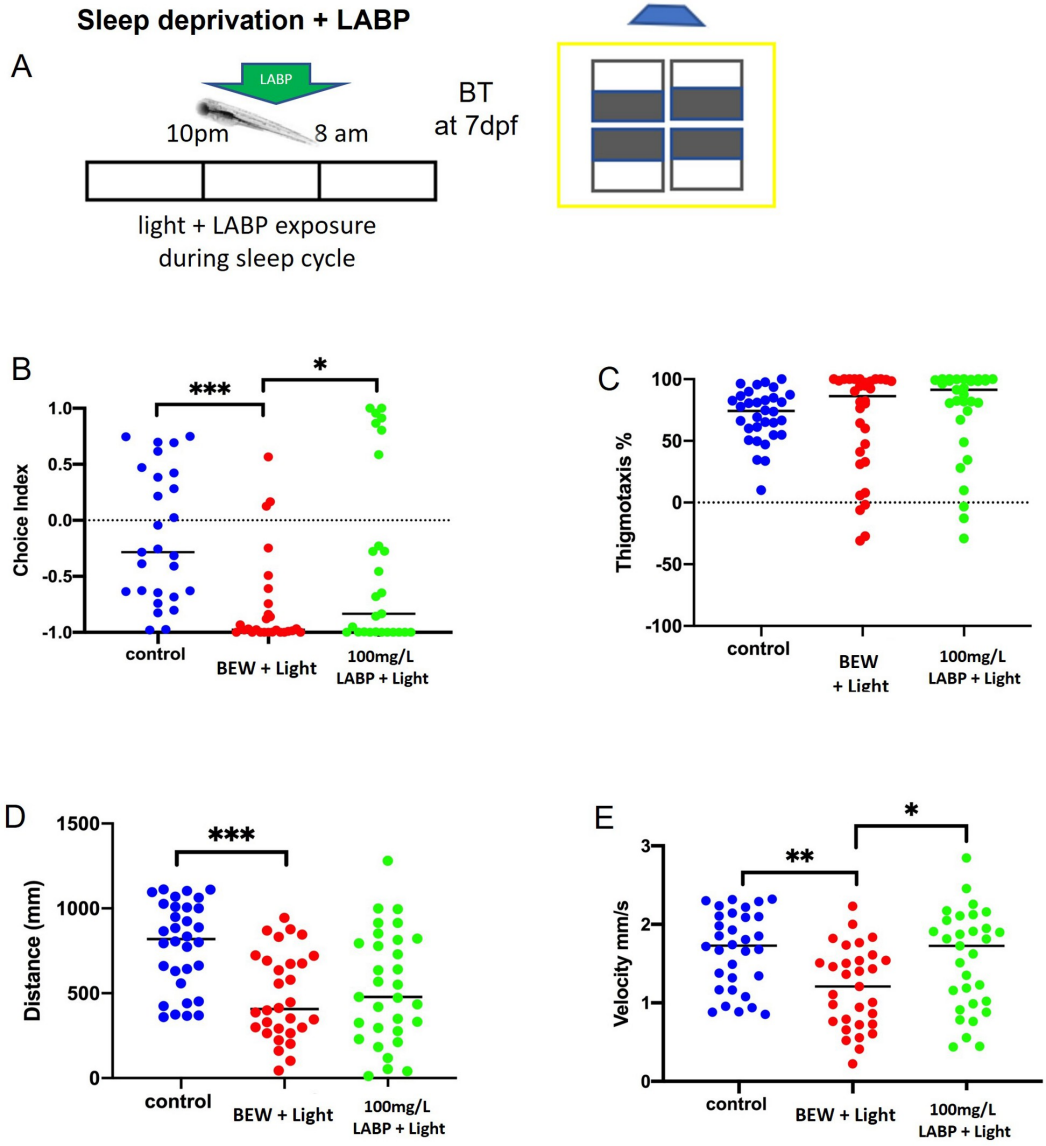
Behavior effects after sleep deprivation and LABP exposure. **A.** The schematic of the behavior test after sleep deprivation + LABP exposure. The larvae were kept on light and LABP exposure during the sleep cycle. **B.** 100 mg/L LABP significantly increased the choice index when compared to control on light (p = 0.0438), while the normal control showed a significantly higher choice index when compared to control on light (p = 0.0009). **C.** Thigmotaxis did not show a significant difference between the groups. **D.** Distance was significantly decreased in control on light groups when compared to normal control (p = 0.0001). **E.** LABP treatment showed significantly high swimming velocity when compared to control on light (p = 0.0228), similar to normal control (p = 0.0015). Asterisks indicate statistical differences between groups (*p < 0.05, **p < 0.01, ***p < 0.001). n = 30/group.

To investigate whether exposure to LABP affects anxiety-like behaviors after sleep deprivation, 6 dpf larvae were subjected to light in the presence of 100 mg/L LABP during sleep hours (Fig 4). The control group was represented by larvae in a normal cycle (kept in the dark between 10 pm and 8 am) in BEW. The choice index was significantly decreased in larvae exposed to light during sleep hours, indicating increased dark avoidance. However, the experimental group exposed to light in the presence of LABP had a significantly higher choice index than the control group (p = 0.0438) (Fig 4B). Thigmotaxis was not significantly affected by these exposures (Fig 4C). The distance traveled and swimming velocity significantly decreased after sleep deprivation compared to the control group (p = 0.0001 and p = 0.0015, respectively). LABP exposure significantly increased the velocity compared to the control group (p = 0.0228) (Fig 4D and 4E).

#### Dark avoidance and thigmotaxis in the presence of a heat stressor can be significantly alleviated by LABP exposure

**Fig 5:**
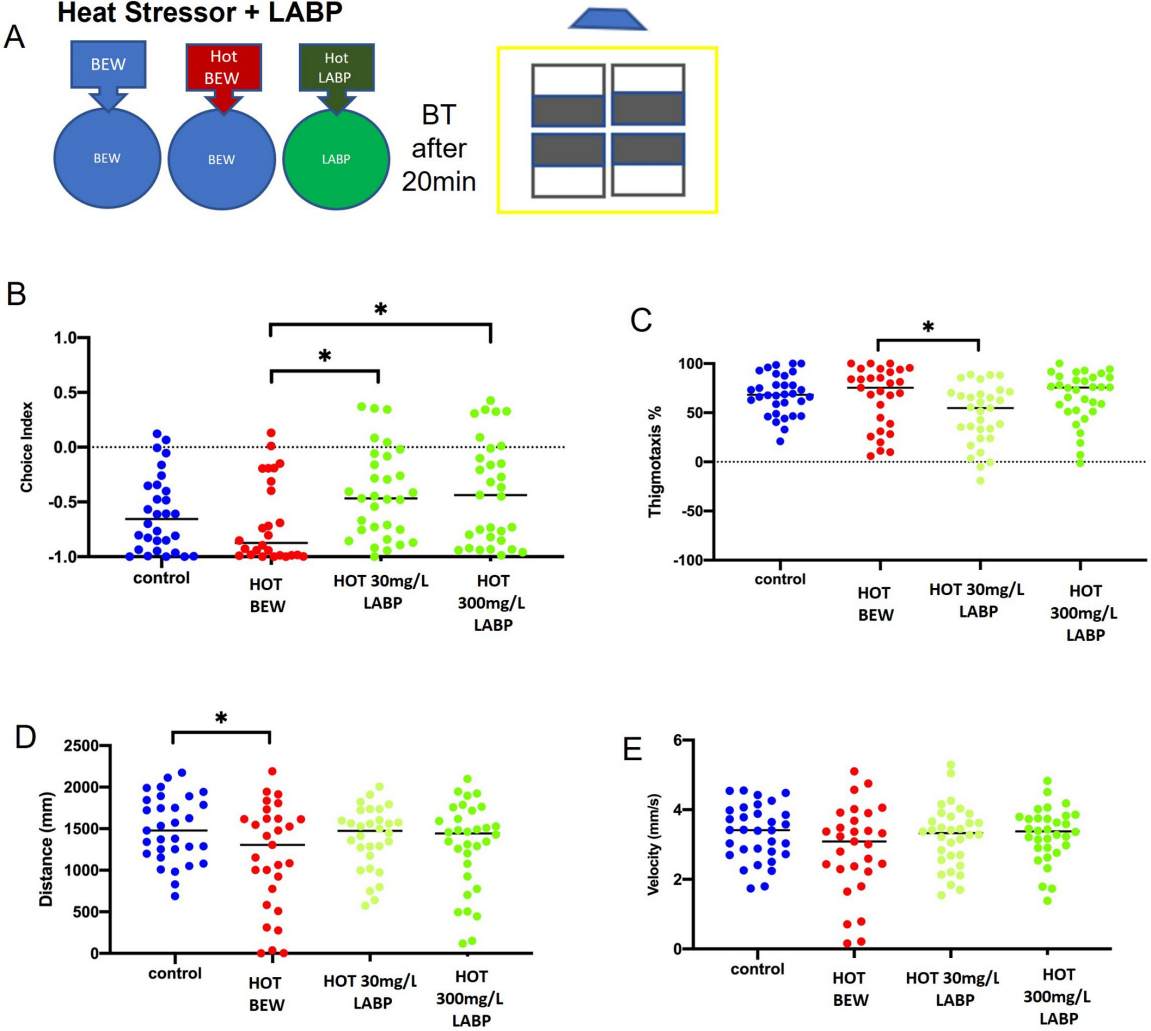
Behavior effects after heat stressor and LABP exposure. **A.** The schematic of the behavior test after the addition of a heat stressor + LABP exposure. **B.** 30 and 300 mg/L of hot LABP significantly increased the choice index when compared to hot BEW (p = 0.0473 and p = 0.0348, respectively). **C.** 30 mg/L of hot LABP significantly decreased the thigmotaxis when compared to hot BEW (p = 0.0314). **D.** There was a significant difference in distance between BEW at room temperature and hot BEW (p = 0.0334). **E.** There was no significant difference in velocity between the groups. Asterisks indicate statistical differences between groups (*p < 0.05). n = 30/group.

To verify the effect of LAPB in the presence of a heat stressor, a behavioral test was conducted at 50°C as described above (Fig 5). The choice index significantly increased after exposure to both 30 and 300 mg/L LABP at 50°C compared to the hot BEW group, indicating decreased dark avoidance (p = 0.0473 and p = 0.0348, respectively) (Fig 5B). Larvae treated with 30 mg/L LABP at 50°C significantly decreased thigmotaxis compared to the hot control group (p = 0.0314) (Fig 5C). The hot control group only showed a significant decrease in the distance traveled when compared to the control group at room temperature (p = 0.0334) (Fig 5D), but the distance and velocity did not show any significant difference between the groups (Fig 5E).

## Discussion

In this study, the behavioral effects of water-extracted LPB on ZF larvae were examined, whereas most of the previous *in vitro* and *in vivo* studies have examined the effects of BP on ethanol or methanol extracts in mice or rats [15–18]. The crude extract of LBP is mainly composed of cinnamic acids and flavonoids [4, 5]; therefore, the characterization of the chemical profile of LBP is an important step in determining the quality of the extract. Here, the chemical composition of the LABP sample was verified by UPLC/DAD-ESI/HRMS/MS, and flavonoids and traces of bufadienolides were identified. Flavonoids have been shown to prevent neurological disorders [23]. Tatsimo *et al*. [24] showed that kaempferol rhamnosides derived from BP possess antioxidant properties. Kumar and Goyal [25] showed significant neuroprotective effects of quercetin in mice with immobilized stress, with altered behavioral and biochemical parameters, suggesting that this compound can be used in stress and related disorders. In ZF, two flavonoids (hesperidin and naringin) showed protective effects, relieving behavioral alterations and morphological defects after alcohol exposure [26]. Hritcu *et al*. [27] reviewed the antidepressant properties of several flavonoids that promote antidepressant-like effects by altering behavior, cytokine levels, oxidative stress, and energy metabolism parameters. It has been suggested that oxidative stress is an important factor contributing to psychosocial disorders.

Current approaches have focused on agents that can modulate multiple targets simultaneously, as the use of multi-targeted drugs significantly increases the range of effects, with fewer adverse effects and toxicity. This approach has been used to design medications for atherosclerosis, cancer, depression, psychosis, and neurodegenerative diseases [28]. Herbal medicines are made up of multivalent compounds with a broad spectrum of activity and exhibit anti-inflammatory effects in addition to being effective in specific indications for various diseases [29]. LBP crude plant extract, instead of an isolated compound, can present multi-target effects and better pharmacokinetic parameters such as greater solubility, agent interactions, and modulation of adverse events, as demonstrated in *cannabis* studies [30]. Among the natural compounds, flavonoids have been studied for their multi-target behavior in the CNS and have neuroprotective activity with different mechanisms of action [31]. Quercetin 3-O-α-L-arabinopyranosyl-(1→2)-O-α-L-rhamnopyranoside (**2**), the major compound present in LBP, can be easily extracted using water [32]; however, the effects observed using the crude plant extract are not expected to be due to this compound alone, as the hydrolysis of a range of flavonol and isoflavone glycosides by the enzyme lactase phrorizin hydrolase, found in the small intestine of mammals, makes it difficult to absorb these compounds as a whole molecule [33, 34]. Quercetin affects locomotion and induces deformities in ZF larvae exposed during development [35]. Bufadienolides, which were discovered in trace amounts in our analysis, are considered toxic owing to their cardiotonic effects [36]. However, studies have provided evidence that they possess anticancer, antiviral, and antioxidant properties [22]. It would be interesting to further investigate the therapeutic and toxicological effects of the isolated compounds in ZF embryos.

ZF is an effective model for studying drug effects on the brain because the blood-brain barrier is similar to that of higher vertebrates [37, 38], which is appropriate for characterizing LABP effects in the present study. At approximately 72 hpf, the blood-brain barrier was established [39]. Experiments can be easily performed by adding the drug to ZF larval medium, which is a good stage for monitoring phenotypic responses. Substances can enter the ZF orally, topically, or through the gills, making it difficult to estimate the exact concentrations absorbed into the tissues [40, 41]. Absorption of the compound is also dependent on the period of development; however, it has been suggested that larvae can be used to mimic adult toxicity [42].

In our experiments, LABP affected the behavior of ZF larvae in a dose-dependent manner. Previous studies have shown that other neuroactive substances also have biphasic effects on ZF behavior. Lower doses of picrotoxin, a GABA antagonist, increased locomotor activity under dark conditions, and higher doses induced a reversal in ZF larvae [11]. A similar concentration-dependent activity pattern was observed following the administration of venlafaxine, a serotonin-norepinephrine reuptake inhibitor [43], in addition to ethanol, d-amphetamine [44], and GABA targets [45]. Generally, stimulating substances, such as adrenaline, increase locomotor activity, whereas sedatives, such as tricaine [11], diazepam, fluoxetine [46], and citalopram (a selective serotonin reuptake inhibitor–SSRI) [47] reduce it.

Previous studies have demonstrated that anxiolytic drugs decrease dark avoidance, whereas environmental stressors increase it in ZF larvae [48]. Fontana *et al*. [49] discussed an important point related to ZF behavior: the trend to explore more “dangerous” areas when less anxious, suggesting that changes in dark aversive behavior are linked to anxiety. From a translational perspective, this has many implications for learning more about anxiety mechanisms. In addition to dark aversive behavior, locomotor activity can also reveal new information. Analysis of locomotor activity demonstrated that exploratory behavior was significantly affected by LABP exposure. Swimming velocity increased after acute LABP exposure during the sleep cycle. The opposite effect was observed after the administration of a higher dose (500 mg/L), resulting in a decrease in distance and velocity. Locomotion, a complex behavior produced by the CNS, plays an important role in animal survival. Therefore, an increase in the level of stress/anxiety can manifest as an increase in the levels of locomotor activity observed during light-dark transitions [11]. Reduction of exploration and increased avoidance are considered ZF phenotypes for increased anxiety/fear-related behavior, whereas reduced activity is related to mood and depression [9]. Our results regarding increased locomotor activity following LABP administration could be interpreted as an improvement in larval energy, a positive effect, and not anxiogenic, because an increased choice index was also observed after LABP exposure, especially after stressor experiments. Elegante *et al*. [50] reported that increased exploration may indicate anxiolytic effects.

Stress represents a threat, potential, real, recognized, or perceived by different regions of the brain, which causes immediate changes in behavior. The stressor is any disturbance of an individual’s environment, which results in effects mediated by neurotransmitters (such as noradrenaline and serotonin), peptides (e.g., corticotropin-releasing hormone [CRH]), and steroid hormones to adapt the animal to the threat [51]. Chen and Baram [52] studied how early life stress programs cognitive and emotional brain networks and cited the high biological significance of the stress to changing situations. Bai *et al*. [48] identified several environmental stressors that significantly affect light preference in larval ZF, including heat. In this study, we used heat as an environmental stressor to verify whether LABP could relieve stress responses. LABP has shown protective and rescue effects following heat stress. Stress response and inflammation are triggered to eliminate stressors, promote adaptations, and restore homeostasis in biological systems [53]. BP is used in traditional medicine as an anti-inflammatory agent [5], and quercetin 3-O-α-L-arabinopyranosyl-(1→2)-O-α-L-rhamnopyranoside (**2**), the major compound, and bufadienolides have also been reported as anti-inflammatory compounds in previous studies [34, 54]. Thus, it is possible to connect the ethnopharmacological synergy information of the crude extract with our results.

Sleep deprivation was another stressor examined by administering light to larvae during the sleep cycle. ZF larvae have sleep-like states similar to mammals and have been shown to have a severe reduction in sleep when kept under constant light conditions [55, 56]. Prober *et al*. [57] demonstrated robust locomotive sleep/wake behaviors at approximately 5 dpf and overexpression of the neuropeptide hypocretin in ZF larvae. The circadian clock organizes several physiological and behavioral functions, including interactions with metabolic and immune systems, beyond sleep and arousal [58]. Learning, memory, attention, anxiety, depression, autism, and schizophrenia are important behavioral processes related to sleep and arousal regulation [59]. Studying sleep and sleep-related behavioral processes can provide insights into new treatments for neurological and emotional disorders. The intensity of voluntary locomotor activity can characterize the arousal state [58]. In this study, an increase in locomotor activity was observed after LABP administration, suggesting that LABP is a partial stimulant, but without increasing anxiety, as demonstrated by a simultaneous decrease in dark aversion.

In rats, methanolic LBP caused a significant reduction in exploratory behavior, loss of residual curiosity, and significant analgesic activity [60]. In mice, LBP also prolonged the onset and duration of pentobarbitone-induced hypnosis, reduced exploratory activities, and delayed the onset of convulsions [61]. In Wistar albino rats, LBP induced an increase in antioxidant status and inhibition of acetylcholinesterase activity, showing an improvement in learning memory [62]. Hosomi *et al*. [18] treated pregnant Wistar rats with BP mother tincture (MT), and while no macroscopic fetal malformations or deaths were observed, high doses of BP produced higher maternal weight gain, suggesting an influence from the orexin/hypocretin system as well as a connection to wakefulness and sleep. In humans, BP significantly improves the subjective quality of sleep and decreases the number of wake-ups and tiredness during the day [63]. Interestingly, in the present study, LABP decreased anxiety-like behavior the day after exposure during the sleep cycle in ZF larvae, which may be due to the improvement in sleep quality, an effect observed in cancer patients treated with BP [64]. Further studies should explore the mechanisms underlying the connection between LABP, anxiety, and sleep quality.

Not all disease-related phenotypes can be identified in ZF, but many developmental and signaling pathways of several diseases are conserved in both ZF and humans [65]. All the above factors analyzed together can display the overall psychoeffects of LABP treatment, which appears to decrease anxiety-like behavior without sedative or depressant effects, including sleep and energy improvements and stimulation without causing agitation. Future studies will aim to examine the mechanisms underlying LBP-mediated behavioral effects, including CNS pathways, neuronal populations, genes, and neurotransmitters.

## Conclusion

Our results indicate that the LABP has an important effect on the behavior of ZF larvae, such as decreasing avoidance and increasing exploratory behavior after different periods of exposure in normal and stressful situations. LABP decreases anxiety-like behavior under stressful environmental conditions without sedative or depressant effects, and we found a stimulatory effect of LABP in larval ZF. This is the first time that these effects have been observed and may be caused by the synergy between the entire components of LABP, mainly flavonoids, known for their anxiolytic and antioxidant properties. Future research to discover the effects of individual LBP components compared to whole extracts, followed by molecular biology studies, must confirm and/or improve our findings.

## Supporting information

S1 Fig Mass spectra (MS/MS) of detected compounds. **2**, quercetin-3-O-α-L-arabinopyranosil (1→2) α-L-rhamnopyranoside; **4**, kaempferol-3-O-α-L-arabinopyranosyl (1→2) α-L-rhamnopyranoside; **5**, kaempferol 3-O-rutinoside; **6**, bryophilin A; **7**, bersaldegenin-3-acetate; **8**, bersaldegenin-1,3,5-orthoacetate.
(JPG)

S2 Fig LABP caused embryo-larval toxicity after 120 h of exposure. ZF larvae were exposed to increasing concentrations of LABP for 120 h. Animal death was determined by examining larvae using a magnifying glass. Larvae that did not have a heartbeat were considered dead. The graph represents four biological replicates (mean ± SEM); each experiment had ten analytical replicates.
(JPG)
