# Supplementary figures and images for: The psychoactive effects of Bryophyllum pinnatum (Lam.) Oken leaves in young zebrafish
Source: PLoS One. 2022 Mar 9;17(3):e0264987. doi: 10.1371/journal.pone.0264987 (PMC8906576; doi:10.1371/journal.pone.0264987)

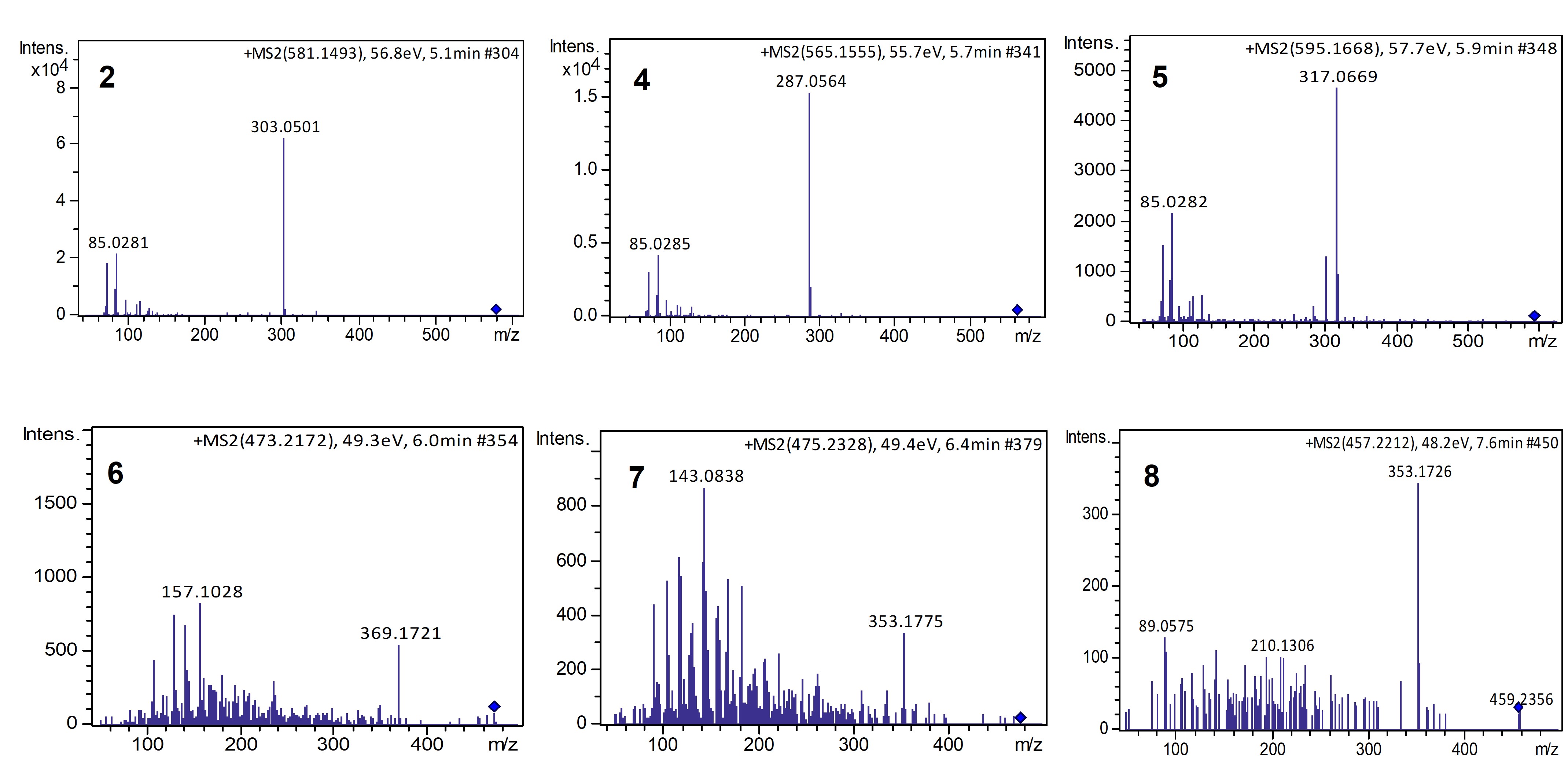

Supplement: S1 Fig — 2, quercetin-3-O-α-L-arabinopyranosil (1→2) α-L-rhamnopyranoside; 4, kaempferol-3-O-α-L-arabinopyranosyl (1→2) α-L-rhamnopyranoside; 5, kaempferol 3-O-rutinoside; 6, bryophilin A; 7, bersaldegenin-3-acetate; 8, bersaldegenin-1,3,5-orthoacetate. (JPG) [file pone.0264987.s001.jpg]

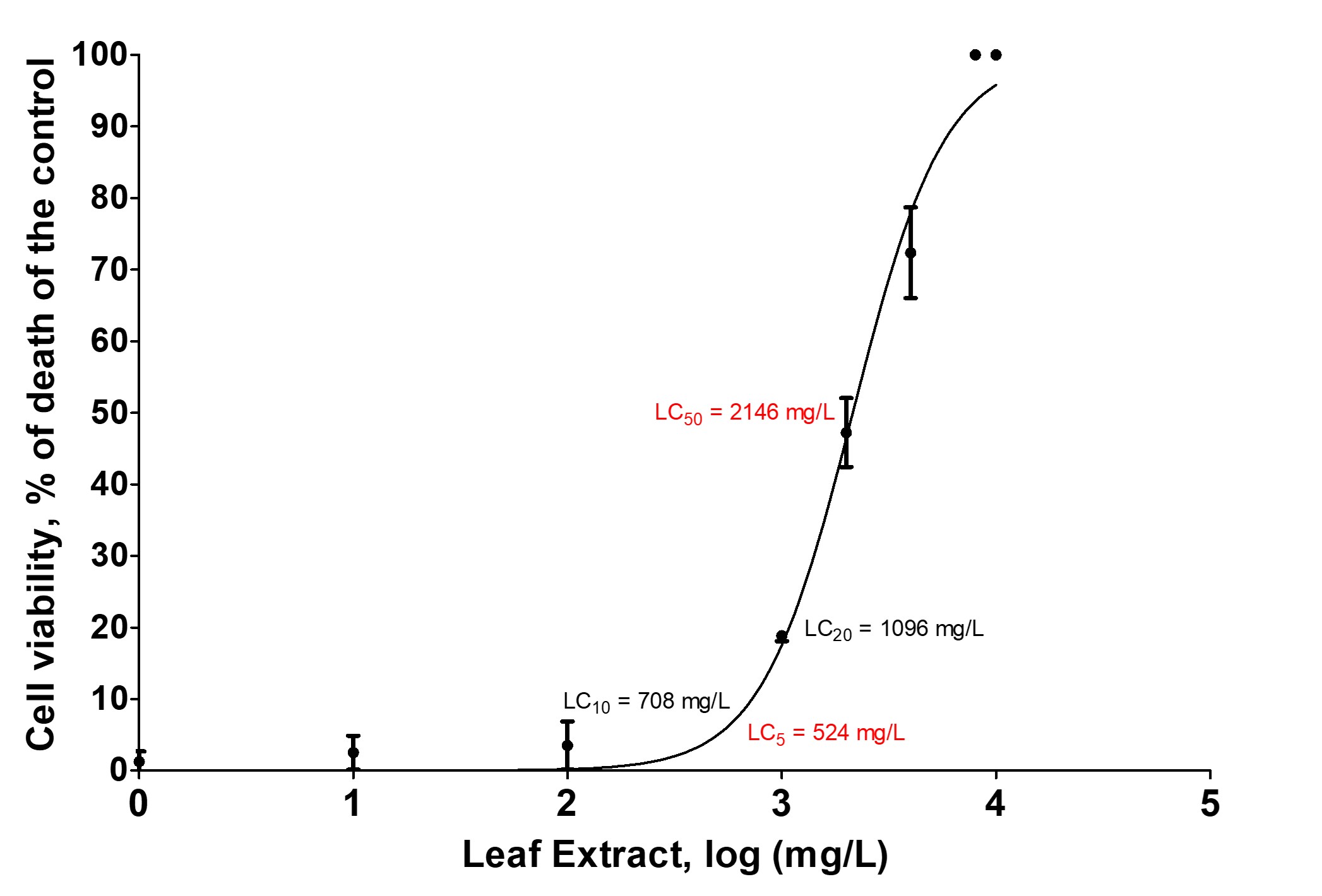

Supplement: S2 Fig — ZF larvae were exposed to increasing concentrations of LABP for 120 h. Animal death was determined by examining larvae using a magnifying glass. Larvae that did not have a heartbeat were considered dead. The graph represents four biological replicates (mean ± SEM); each experiment had ten analytical replicates. (JPG) [file pone.0264987.s002.jpg]
